# Food and Nutrition Champions in Residential Aged Care Homes Are Key for Sustainable Systems Change within Foodservices; Results from a Qualitative Study of Stakeholders

**DOI:** 10.3390/nu13103566

**Published:** 2021-10-12

**Authors:** Danielle Cave, Karen Abbey, Sandra Capra

**Affiliations:** School of Human Movement and Nutrition Sciences, Faculty of Health and Behavioural Sciences, The University of Queensland, St Lucia, QLD 4072, Australia; k.abbey1@uq.edu.au (K.A.); s.capra@uq.edu.au (S.C.)

**Keywords:** food fortification, foodservices, aged care, older adults, implementation

## Abstract

The role of foodservices in aged care is difficult to understand, and strategies to improve the nutritional care of residents are often unsustainable. In particular, food-first strategies such as food fortification are poorly executed in everyday practice and its execution relies upon the foodservice system in aged care homes. The aim of this study was to explore the perspective of staff on the role of foodservices in aged care and gauge the level of skills, education, access, time, and ability to deliver food fortification. Semi-structured interviews were conducted with foodservice managers, foodservice workers, dietitians, carers, and other managers who work in aged care homes across Australia. Participants were recruited purposively through email and through snowballing. Interviews (*n* = 21) were recorded, transcribed verbatim, and analyzed using inductive thematic analysis. Three themes and six sub-themes were identified. The three themes include the role of foodservices being more than just serving food, teamwork between all staff to champion nutrition, and workplace culture that values continuous improvement. These themes identify how staff perceive the role of foodservices in aged care and provide an important perspective on the long-term sustainability of food fortification strategies and how to improve current practice.

## 1. Introduction

For more than 20 years, the residential aged care sector in Australia has been highly criticized. The most recent national enquiry was a Royal Commission that reported in 2021, which was critical of a range of issues around food and nutrition, including the failure to manage the rate of malnutrition, the requirement for increased choices, the need for person centered approaches and major issues with staffing and skills [1]. As policies supporting aging in place at home become accepted practice, those requiring residential aged care are now older, frailer, and require smaller and nutrient dense meals. Anecdotally, sustaining food fortification strategies in aged care foodservice systems in Australia is difficult. It has been postulated that the high turnover of staff, exacerbated by the low pay of Australian aged care workers and an aging workforce have been contributors. Workforce is an important issue because as the population ages, so does the aged care workforce. In Australia, over the next decade it is expected that demand for staff, particularly qualified staff in this sector will overtake supply and it is estimated that the sector will need 110,000 additional staff [2]. Workforce shortages in aged care will continue to be a significant challenge in Australia, with predictions that by 2050 there will be a shortage of 980,000 staff in the aged care sector [3]. Pay for aged care workers starts at $AUD21.62 per hour and is just above the minimum wage level in Australia, which in 2021 is $AUD20.33 per hour [4,5]. A Royal Commission into Aged Care Quality and Safety was established in October 2018 as a national enquiry into the aged care sector. The scope of this Royal Commission included the challenges that will arise in the future given the demographic changes expected, how the Government can improve the system, how to create an aged care system which is consumer-oriented allowing for choice and control for residents and users, and how services can be delivered sustainably, with the use of technology and investment in the workforce [1]. Low pay, limited opportunities for career progression, and limited access to ongoing training have all been listed as negative perceptions of working in the sector [1,6]. This contributes to staff instability, which can subsequently destabilize the delivery of nutrition support strategies including food fortification.

In Australia, tertiary education includes higher education and vocational education and training (VET) [7]. VET qualifications are provided by either government or private institutions and include four levels of certificates (Certificate I, II, III and IV), Diploma, and Advanced Diploma courses. In 2016, it was reported that 90% of aged care workers held post-secondary qualifications and over two-thirds (67%) of personal care assistants held a Certificate III in Aged Care, which is the common qualification for this position [3]. Whilst foodservice staff typically hold a qualification, it is acknowledged that formal training of commercial cooks has no content relating specifically to the aged care sector [8]. In response to recommendations in the Aged Care Workforce Strategy, the Aged Services Industry Reference Committee (IRC) are developing the Residential Aged Care Cookery Skill Set, which targets cooks and chefs that hold a Certificate III in Commercial Cookery (or equivalent) and are seeking further skills and training to work in residential aged care homes [6,9].

Aged care staff have raised concerns that they receive inadequate training to manage residents now entering aged care with more complex conditions [1]. Additionally, staff may have little knowledge of the nutritional requirements of aged care residents and may require additional training to address knowledge gaps [10]. Public health nutrition measures such as dietary guidelines are not appropriate for this population, where the main concern is adequate protein and energy intake [11]. Malnutrition remains an ongoing concern in Australian aged care homes, with reports that up to 50% of residents can be malnourished or at risk of malnutrition, although the literature is lacking recent data on malnutrition rates in this setting [12,13]. This has remained stubbornly high for more than two decades and requires better management. The challenge is to provide high protein, high energy, high nutrients in small, easily consumed meals. Food fortification is a food-first nutrition support strategy and involves the addition of nutrient-dense ingredients such as cream, milk powder or a supplement powder to food and beverages, without increasing the portion size [14]. This strategy can be implemented across the menu and is effective for residents who are small eaters. There are currently no best practice guidelines for the delivery of food fortification in this setting and the Aged Care Quality Standards are silent, leading to practices varying between homes [15,16].

Foodservices in aged care play a critical role in the nutritional care of residents and there is a need for sustainable strategies that an aged care home can implement and maintain that will reduce or ameliorate malnutrition long term. Food fortification is a versatile strategy where a variety of ingredients can be added to many foods and beverages across the menu. There are many potential issues in implementing and maintaining food fortification strategies including the inflexibility of foodservice systems and strict budgetary restrictions, which ultimately can limit the strategies which can be implemented [10]. Foodservice systems need to meet the needs of a large number of people and are not always able to meet individual dietary preferences. Key success factors include adequate resources such as skill, education, access, time, availability, and low staff turnover. Additionally, dietitians in Australia are rarely employed by aged care homes, which are primarily (91%) run by not-for-profit and for-profit private organizations, but instead work as consultants on a referral basis or at scheduled intervals [17,18]. So, whilst they might prescribe strategies such as food fortification, they rely on the onsite foodservice staff to oversee the preparation and delivery of strategies, as well as care staff to ensure the food is consumed. Anecdotally, strategies that are suggested or implemented rarely continue over time.

There is limited literature exploring how the role of the foodservices has changed in residential aged care to manage the changing clientele. Additionally, there is little exploration of the opinions of key staff in aged care homes around food fortification strategies; an important issue for implementation and long-term sustainability. Only one study has been identified that used qualitative methods with key stakeholders, including staff in aged care homes in Canada [19]. This study specifically focused on gathering data on acceptability of micronutrient food fortification strategies, rather than implementation and long-term sustainability. No data was gathered on staff skill, education, access, time, and ability to deliver food fortification. The aim of the present study was to understand the opinions of key staff in Australian aged care homes about food fortification and more broadly, the role of foodservices in aged care and what needs to happen to maintain suggested changes.

## 2. Materials and Methods

A qualitative approach was chosen as most appropriate for this study [20]. Semi-structured in-depth interviews were conducted focused on the questions: (1) what is the perspective of key stakeholders on the role of foodservices in aged care; and (2) do staff have adequate skills, education, access, time, and ability to deliver food fortification? The researchers adhered to the Consolidated Criteria for Reporting Qualitative Research (COREQ) [21]. The University of Queensland provided ethical approval for this study (approval number 2019000008) and informed consent was obtained from all participants.

### 2.1. Sampling

A purposive sample of foodservice workers from residential aged care was recruited. The initial sample consisted of a mix of staff who currently or had recently worked for at least 3 years in aged care homes in Australia. An open email invitation to participate was sent to all members of a mailing list of persons with established relationships to the researchers (*n* = 38). This group consisted of foodservice managers, foodservice workers, dietitians, care staff and other managers. Further participants were recruited through snowballing, as participants were invited to forward the email invitation to their networks. As a result, the total number of people who received an email invitation but declined to participate in this study could not be quantified. The invitees received the Participant Information Sheet and Consent Form (PICF) attached to their email invitation, which outlined the purpose of the study and the background of the female researcher (BNutrSc, MDietSt, PhD Candidate and Dietitian). A single reminder was sent one week after the initial invitation or at a future date as requested by the recipient.

### 2.2. Data Collection

An interview guide was created based on gaps identified by the researchers in a narrative literature review [15]. The interview guide was composed of 28 questions: 3 related to what the interviewees perceived to be the main issues in foodservices, 5 related to unintended weight loss, 4 related to the menu and nutrition support strategies, 9 related to staff responsibilities and training, 3 related to long-term sustainability, and 4 related to barriers to change that may impact the implementation of food fortification strategies. Issues such as unintended weight loss, the menu and its composition, staff responsibilities and other staffing issues were specifically raised during the interviews. The interview guide is summarized in Appendix A. The interview questions were piloted for construct validity on two volunteers, with minor adjustments made prior to study commencement.

Interviews were conducted either face-to-face (at the University of Queensland or the participant′s workplace) or by telephone, depending on the location and preference of the participant. All interviews were conducted from May to August 2019 and recorded using an MP3 audio recorder by one researcher (D.C.). Only the participant and researcher were present during the interviews and no repeat interviews were required. Field notes reflecting on the interview and noting ideas for data analysis were written at the conclusion of each interview. Demographic information was collected during the interview, including age range, gender, education level, occupation, length of employment in the aged care sector and if any training in nutrition had been completed whilst employed in the aged care sector. Participants were informed in writing that they could have their transcript returned to them for feedback, however none of the participants requested their transcript.

### 2.3. Data Analysis

Interviews were transcribed verbatim by an independent transcription service [22]. The transcripts were then checked against the recordings for accuracy by one researcher (D.C.). Transcripts were analyzed using an inductive thematic analysis approach, guided by the six phases of thematic analysis defined by Braun and Clarke [23]. The six phases of thematic analysis include: (1) familiarizing yourself with your data; (2) generating initial codes; (3) searching for themes; (4) reviewing themes; (5) defining and naming of themes; and (6) producing the report. Transcripts were coded by one researcher (D.C.) and labels were applied to elements of the text using the qualitative analysis software NVivo 12 [24]. Codes, themes, and sub-themes were reviewed and further developed through discussion with a second researcher (S.C.). Themes and sub-themes were verified using a subset of five transcripts (25%) by two researchers (D.C. and S.C.).

## 3. Results

A total of 17 of the 38 initially contacted responded (45% response rate), with a further 7 enrolled through the snowballing phase. All came from a variety of homes and locations within Australia. Of 24 who expressed interest, 21 (88%) interviews were completed with a median duration of 30 min (15–50 min). Participant characteristics are summarized in Table 1.

### 3.1. Thematic Analysis

Analysis continued until data saturation was achieved and no new themes were identified. Following inductive thematic analysis, researchers identified three themes and six sub-themes. In terms of the role of foodservices in aged care, there was consensus that there are opportunities to make positive changes in the operation of foodservices through teamwork and the workplace culture. Themes and sub-themes are summarized in Table 2.

### 3.2. The Role of Foodservices Is More Than Just Serving Food

The role of foodservices in aged care encompasses the preparation and serving of food to residents, but this role goes beyond merely the provision of food and overlaps with clinical care. The role of foodservices does not stop once the meal is provided, but also involves monitoring intake of residents and enquiring as to why the meal was not consumed when necessary.

*It should all be part of the foodservice, but often that isn′t either. I think often the foodservice system sort of thinks that its role stops at the point where they′ve provided the meal, rather than looking at what′s been eaten or not eaten, more importantly so and the reasons why that might not have been taken. [#21 Dietitian]*

Each meal was considered to be an opportunity to provide nutrition for residents and impact their quality of life.

*We have the opportunity to impact on every resident′s life six times a day … by providing them nutritious, healthy, beautiful looking tasty food. [#3 Foodservice Manager]*

Taste was considered to be a very important aspect of foodservices, with staff commenting that the food will not be consumed if it does not taste good. Therefore, weight loss could be the result of a foodservice that does not provide food that meets the taste preferences of residents, or that does not monitor consumption of the food provided.

*If something doesn′t taste good, whether you′re losing weight or not, you won′t eat it. So I think the foodservices side kicks in massively on that. Probably over and above the clinical, in that we′ve got to produce a product that tastes delicious, that is well and truly fortified, that they will enjoy eating hence they will eat, and then hopefully gain weight. [#18 Foodservice Manager]*

Foodservices can help meet the needs of most residents through the menu, including the use of food fortification strategies. However, there was a strong opinion that a poorer foodservice can lead to a reliance on commercially available oral nutrition supplements (ONS) to provide additional nutrition support to residents.

*You should be able to do most of it with your foodservice, but your foodservice needs to have the flexibility and the ability with the processes to help the residents before we start making (companies that sell ONS) very happy. [#17 Dietitian]*

#### 3.2.1. Resident Input

It was evident that resident input should be important to foodservices, including in menu planning. However, it was mainly dietitian respondents who noted this more commonly than other staff. Therefore, foodservices need to provide an avenue for residents to regularly provide their input.

*Sometimes I think they′re not listening to the residents enough as to what they would like and getting residents involved in the menu planning could be something also improved. [#15 Dietitian]*

It was not just about what residents would like to see on the menu, but how they would like other aspects of the foodservices to operate such as the presentation and timing of their meals.

*I think also in particular and at the moment, it′s not really collaborating a lot with the patients about what sorts of food they′d like and how they′d like it served and all of that kind of thing, is a big part. [#21 Dietitian]*

*What it is actually is more of an approach on an individual resident basis about what they actually really want and a concentrated effort by foodservices to actually guarantee that … [#7 Manager]*

This would help residents feel like they are able to participate in the operation of their home and that their contribution is valued by staff.

*It just makes them feel like-and that, they are part of that process then is mostly because it′s not just about-if they want to be, it′s not just about being a recipient it′s actually being a participator in their home. [#6 Dietitian]*

#### 3.2.2. Providing Choice

If the foodservice allows resident input in meals, it also helps meet other needs of residents such as providing choice, autonomy, respect and dignity.

*Just that there′s a lot more emphasis on the resident involvement in meals and like I said at the beginning, I think that fills social needs providing choices and autonomy and respect and dignity for the patients (sic). [#21 Dietitian]*

A focus on providing choice to residents has been driven in Australia by the latest Aged Care Quality Standards, which came into effect in July 2019. Standard 4 (3) (f) stipulates that “Where meals are provided, they are varied and of suitable quality and quantity” [16]. This standard expects that meals are based on the consumer′s preferences, which includes religious and cultural considerations.

*… it all comes down to resident choice and the decision—it is their life, after all. That works for care side and food side, as well. [#14 Kitchenhand]*

This has helped improve choice for residents within the aged care sector by requiring homes to provide opportunities for residents to voice their opinion, such as through food forums.

*But most aged care facilities I′ve noticed are going to the food forums. They′re asking the residents. It′s all about the new standards as well, because it′s all about choice, so what is it you want. So, yeah, they have pretty good choices. [#9 Manager]*

### 3.3. Teamwork between All Staff to Champion Nutrition

Dietitians have traditionally been the champions of nutrition, however in settings like aged care where the dietitian is often not based on site and may only visit periodically there is a need for other staff to take on this role. Aged care staff need to work alongside dietitians to champion nutrition within the home.

*A dietitian can′t do it on their own … it should be a team of people really and that′s probably what′s really wrong is that lack of team isn′t it? [#21 Dietitian]*

Rather than selecting a single food and nutrition champion within the home, input from a larger multidisciplinary team would provide more ideas and information. However, it was clear that there is a role for a leader within the team and a need for more consistent champions, not simply periodically.

*I think when you have a multidisciplinary approach … you get better ideas, you get newer, fresher … When you′ve got lots of input, I think it′s far more colorful and creative. So although you have to have someone leading the ship, I think that that leader needs to have input from other people. [#9 Manager]*

All staff across the home need to help advocate for nutrition and communicate with each other when there any issues that need to be flagged.

*I think everyone should be advocating. I think it′s again—it′s a clinical, it′s a foodservices and it′s a case of communication. So everybody should be effectively trying to achieve that goal and I think it′s everybody′s responsibility to do so. [#18 Foodservice Manager]*

*Well I think dietitians should have a say and recommend, and then liaise again with your hotel service manager and your care service manager… [#13 AIN]*

Additional training may need to be provided to staff to ensure that they know their role within the team and how they can champion nutrition for aged care residents. High staff turnover can exacerbate issues.

*We don′t want them to have the supplements as such but it′s also about educating our nurses, how to feed a person that is malnourished. It′s about everybody—we work as a team, we′re not isolated. [#1 Foodservice Manager]*

*The care staff just believe that they have too much else to do, so they bring the residents in and then they run off and do something else … I don′t believe that they work together with us, as much as it should be, for the benefit of the residents. [#19 Kitchenhand]*

#### 3.3.1. Resident-Centered Care

Resident-centered care involves giving residents the opportunity to participate in decisions relating to their care and respecting their individual preferences. There was an emphasis on resident-centered care and that it was the role of the aged care home to meet the needs of residents, particularly when it came to providing nutrition support, in a personalized way.

*So each individual and each resident is completely different as to how you would approach it. I think that′s how it should be. [#3 Foodservice Manager]*

The majority of interviewees were not restricted by cost when providing nutrition care to residents.

*We don′t have any (cost) restrictions here. If someone needs something, they will get it. [#12 Foodservice Manager]*

*… (costs)... are not important at all. We don′t give a turkey how much it costs, as long as that person′s happy. [#9 Manager]*

*Cost—yeah, definitely less important … I don′t know about other aged cares (sic), but definitely the top priority here is their wellbeing and not the cost of different things [#10 Kitchenhand]*

#### 3.3.2. Food-First

Using a food-first approach to manage malnutrition, including food fortification strategies were the preferred approach amongst interviewees and the successful implementation involved teamwork between dietitians and foodservice staff.

*Yes, I′ve worked as the foodservice dietitian for one group in aged care, and yes, we—and in fact a few groups now, we′ve implemented a high energy, high protein diet code which was basically a fortified... [#17 Dietitian]*

*As I say, I work with a dietitian and we used to—we had 1,300 residents over 16 sites and we used to monitor their weights, we used to make weekly and we used to fortify sauces, soups, custards, all those sorts of things to try and encourage them to get more calories. [#11 Foodservice Manager]*

Teamwork between all staff helps in the implementation of the food-first approach within aged care, which can be difficult to achieve when driven by a dietitian that is predominately based offsite.

*I think we can—we′re there to help maintain it, and I have a food first philosophy. I think the food-first philosophy is actually complicated in aged care to actually achieve that because it requires so much input at so many levels, which is probably why there′s such an over-reliance of (ONS). [#17 Dietitian]*

This approach was also considered to be less expensive than relying on ONS.

*So you′ve got your food first approach. I think when management of these organizations realize the cost of food versus supplements and medication, I think when they do the math′s, they will actually work out that it′s a lot cheaper for them to use the food first approach, rather than to just automatically put someone on a supplement when you see that weight loss. [#3 Foodservice Manager]*

### 3.4. Workplace Culture That Values Continuous Improvement

Workplace culture was perceived to be both an enabler and barrier to making positive changes within the home.

*Culture and attitudes, biggest barrier. Especially if you′ve got a working site where they′ve had a lot of staff there for a long period of time. But you can make the change, you′ve just got to chip away, chip away, chip away. [#11 Foodservice Manager]*

If there is a culture of continuous improvement within the home, then staff understand that change is normal and necessary.

*You have a culture in a home of continuous improvement, of quality assurance, and everyone knows that those processes that they use every day and they understand those processes and why they′re important and they′re all active in them, then not only do you have a safe work environment, but you′re continuously improving. [#2 Manager]*

#### 3.4.1. Leadership Drives Change

Strong leadership drives change and helps support the foodservice system, therefore foodservices can have difficulties when managers lack leadership skills. Leadership is also required to ensure implementation and continuation of change.

*Probably lack of leadership in people higher up in management driving that change, because I think without their support it′s very hard for foodservices to make a change. [#15 Dietitian]*

*I′ve got some training I′m meant to be doing, but I′ve been meant to be doing for like a wee while now and it just hasn′t happened … I guess that′s the boss′s responsibility to make sure these things get done… [#10 Kitchenhand]*

Managers that provide strong leadership have a positive workplace culture and teamwork amongst their staff.

*It′s really dependent on the manager. If that manager runs a really good home and really gets their culture and that team working, it′s just magic. [#2 Manager]*

It was also felt that aged care homes that has strong leadership experienced lower rates of staff turnover.

*In well-run facilities, no. In poorly run facilities, absolutely yes. [#7 Manager]*

#### 3.4.2. Training Opportunities for Staff

Staff training is an investment and may help keep staff employed in the aged care sector, where staff turnover is known to be high.

*You can talk about it but the proof′s in the pudding. Staff I think—people—will respond to an environment where you do make an investment in them and you do recognize their performance, and you do allow them to make a mistake, and if they can see a career path. [#8 Manager]*

Staff training should not be seen as an opportunity for cost savings within the home, but as an investment in staff.

*I think that a lot of times in aged care the short-term cost of training and getting people and all that, they don′t see the long-term benefits, if that makes sense. [#4 former Foodservice Manager]*

More training is needed to upskill the aged care workforce, including for foodservice and care staff.

*I feel the other thing is the lack of skilled workforce in foodservice … there′s just no training from the foodservice to the carers to the table really. [#6 Dietitian]*

Additionally, it was felt that staff are interested in learning and attending training, but there are not always opportunities for them to do so.

*Some of them would dearly love to understand more and have more appropriate training. [#6 Dietitian]*

*We do like a mandatory training that we do the basic stuff like fire safety and health and safety and things like that, but I definitely think we could do with some more. [#10 Kitchenhand]*

## 4. Discussion

This study explored aspects of aged care foodservice from the perspective of stakeholders within the system as well as external consultants. It used semi-structured interviews and thematic analysis. Themes identified were the role of foodservices is more than just serving food, teamwork between all staff to champion nutrition and workplace culture that values continuous improvement. These are new to this context, with no prior literature that the authors could find specific to aged care homes. There was no universally held position on the role of foodservices in aged care. The role of foodservices is more than just serving food and requires support from both foodservice and care staff to ensure that residents are not just receiving, but are consuming the food. Whilst there was a general understanding of the issues currently facing foodservices, there were few solutions nominated in how to rectify the issues raised through either the systems in which respondents were working, those raised in the Royal Commission or as a result of the Aged Care Quality Standards [1,16].

The interviews highlighted there was tension between care and foodservice staff around work tasks and not undertaking each other′s role. It was difficult to recruit care staff to participate in this study, as the majority of care staff declined to participate and commented that foodservice was not part of their role, despite their role being critical to the success of consuming meals. Snowballing strategies also failed to recruit any additional care staff.

*The interview was very short compared to others, the AIN did not want to participate outside of work hours but was very busy. The interviewee did not know a lot of the ins and outs of foodservices, however did discuss aspects of teamwork and interacting with the foodservices manager. [Fieldnotes, 22 May 2019]*

Both care and foodservice staff have an essential role to play in the provision of food and nutrition within a home and ensuring residents have input and are provided with true choice (providing meaningful options that meet residents′ preferences) [25]. The strength of food fortification is that it can be tailored to residents′ food preferences. The Aged Care Quality Standards, particularly Standard 4 (3) (f) require homes to provide evidence of their processes to deliver nutrition consistent with residents′ needs and preferences, as well as how residents are involved in menu planning [16]. However, while this was not reported as happening in practice, it was identified that the role of foodservices was more than just serving food and that all staff can have a role to play in championing nutrition.

Dietitians working in the aged care sector need to work as part of a team with both care staff and foodservice staff to champion nutrition and advocate for a strong and explicit focus on resident-centered care. Dietitians in the systems studied here had many comments but were not present at aged care homes daily or even weekly to champion change, as they usually operated as external consultants. This was a major issue which appeared to impact their issues and concerns, with their focus on clinical issues rather than systems issues. It is difficult to achieve teamwork within the aged care home when there are no dedicated staff members embedded within the system, who are present onsite each day to champion food and nutrition. The findings of this study further support a mandate for identified food and nutrition champions, and making nutrition everyone′s business [10,26]. This conclusion is strengthened by the finding that despite the call for champions for more than a decade they are still not common within the system; only one of the homes involved in our study had them in place [10]. Additionally, building a workplace culture that supports staff to work towards shared goals can help staff better understand their and others′ roles and support teamwork [27]. This could benefit both care and foodservice staff to understand their roles, provide support to the dietitian and support the delivery of resident-centered care [28]. These changes would support the delivery and ongoing monitoring of food fortification strategies using a team approach and embed food fortification within the foodservice system.

Workplace culture was perceived to be both an enabler and barrier to change. Failure to change and improve current practice and workplace culture is not conducive to long-term sustainable improvements with the aged care sector. These findings are in agreeance with Recommendation 89 of the Royal Commission into Aged Care Quality and Safety where from July 2021, aged care providers must “adopt and implement a plan to manage and support staff training, professional development and continuous learning, staff feedback and engagement, and team building” [1]. Providing access to ongoing training has been considered to be a positive organizational value in this sector, although funding constraints have been noted as a reason for less frequent training days, with training moving towards online modules or short presentations [3]. Aged care staff that feel well supported with regular training feel motivated and training assists with the development of skills [29]. Aged care providers need to ensure that there is strong leadership driving change within aged care homes at the systems level, which would be further supported by food and nutrition champions.

The limitations of this study include the lack of generalizability of findings globally, as only Australian aged care workers were interviewed. There may also be selection bias present, as those who agreed to be interviewed may have been more interested in nutrition and improving foodservices. There was difficultly recruiting care staff who did not feel like they could comment on the role of foodservices, and they may have provided a different perspective. No recommendations could be made from the findings of this study relating to whether staff have the skills, education, access, time, and ability to deliver food fortification specifically. However, sub-themes were identified relating to providing training opportunities for staff and staff preferred a food-first approach to nutrition support in this setting. Whilst there was a relatively small sample size, data saturation was met and no further themes could be identified.

## 5. Conclusions

This study confirms that the role of foodservices within aged care goes beyond the provision of food to residents. It also confirms that while nutrition champions have been seen as critical for many years, they still do not exist in the majority of homes. Lack of training and staff turnover are potential outcomes of this deficiency. In order to make long-term sustainable changes in foodservices, a team with an identified leader should be established to champion nutrition. There should be a strong and explicit focus on resident-centered care using a food-first approach. Additionally, workplace culture needs to value continuous improvement, which is driven by strong leadership and providing training opportunities for staff. The themes and sub-themes identified in this study demonstrate how staff perceive the role of foodservices in aged care and provide an important perspective on the long-term sustainability of strategies and how to improve current practice and manage malnutrition. It is clear there is a need for change at the systems level if the issues within aged care foodservices are to be resolved as it cannot be on a home-by-home basis.

## Figures and Tables

**Table 1:** Participant Characteristics.

Characteristic	*n* = 21
Gender, *n* (%)	
Male	5 (24)
Female	16 (76)
Age group in years, *n* (%)	
25–34	1 (5)
35–44	4 (19)
45–54	9 (43)
55–64	5 (24)
65–74	2 (9)
Highest level of education ^a^, *n* (%)	
Postgraduate Degree	3 (14)
Graduate Diploma and Graduate Certificate	1 (5)
Bachelor’s degree	6 (29)
Advanced Diploma and Diploma	3 (14)
Certificate III/IV	5 (24)
Year 12	1 (5)
Year 11 or below (includes Certificate I/II)	2 (9)
Occupation ^b^, *n* (%)	
Dietitian	4 (19)
Carer (AIN)	1 (5)
Kitchenhand	3 (14)
Foodservice manager	9 (43)
Manager (ACFI coordinator, general manager, non-executive director & CEO)	4 (19)
Years of employment in the aged care sector, median (range)	12 (4–38)
Training in nutrition during employment in the aged care sector, *n* (%)	
Yes	16 (76)
No	5 (24)

^a^ Highest level of education has been ranked according to the Australian education system, from tertiary education (higher education (Bachelor Degree to Postgraduate Degree) and vocational education and training (VET) (Certificate III/IV to advanced diploma)) to secondary school (years 11 to 12) or below [7]. ^b^ AIN = assistant in nursing; ACFI = Aged Care Funding Instrument; CEO = chief executive officer.

**Table 2:** Themes and Sub-Themes.

Themes	Sub-Themes
The role of foodservices is more than just serving food	Resident input
	Providing choice
Teamwork between all staff to champion nutrition	Resident-centered care
	Food-first
Workplace culture that values continuous improvement	Leadership drives change
	Training opportunities for staff

## Data Availability

All interview transcripts are held in secure storage by the University of Queensland and are not publicly archived, but available upon approved requests.

## Appendix A

Interview Guide:

Demographic information

- What is your age?
- What is your gender?
- What is the highest degree or level of school you have completed?
- What is your current occupation?
- How long have you been employed in the aged care sector?
- Have you undertaken any training in nutrition whilst employed in the aged care sector?

Main issues in aged care

- Can I talk to you about what you think are the main issues in aged care foodservices?
- What do you see as the primary role of foodservices in aged care?
- What is your opinion about the Royal Commission in the aged care sector? Will it address these main issues?

Unintended weight loss

- What are your thoughts around residents losing weight in aged care?
- What do you think the role of the foodservices is in treating weight loss?
- Which residents do you think we could help?
- Do you think everyone should be treated or when would you intervene?
- What are your experiences trying to stop weight loss leading to malnutrition?

The menu

- When do you think is the best opportunity in the day to target residents who are not eating much?
- How important are costs when considering a solution to unintended weight loss?
- Have you tried food fortification or another food-based strategy as a solution?
- Would you buy in pre-made food fortification products if you could?

Staff responsibilities

- What are the tasks in foodservices that take up the most time?
- Is there enough time to meet the needs of residents?
- Do you have enough time for staff training in foodservices?
- Can you keep up with staff training requirements for new staff?
- What are the barriers to training staff?
- Do you have a high foodservice staff turnover?
- Do care staff understand the role of foodservices?
- Do care staff support foodservices?
- Who runs the dining room?

Sustainability

- In your opinion, who should champion the nutritional care for residents?
- What might be the issues with allocating a person to act as a nutrition champion?
- Can you give an example of something you have seen changed or improved within foodservices and who championed this change or improvement?

Barriers to change

- Can you think of an example of a task that has been started in foodservices but then stopped?
- Why do you think it stopped?
- Can you explain what you think is the biggest barrier to change in foodservices?
- Can you think of any solutions to this barrier?
